# Fatal food anaphylaxis in adults and children

**DOI:** 10.1186/s13052-024-01608-x

**Published:** 2024-03-05

**Authors:** Elio Novembre, Mariannita Gelsomino, Lucia Liotti, Simona Barni, Francesca Mori, Mattia Giovannini, Carla Mastrorilli, Luca Pecoraro, Francesca Saretta, Riccardo Castagnoli, Stefania Arasi, Lucia Caminiti, Angela Klain, Michele Miraglia del Giudice

**Affiliations:** 1 Allergy Unit, Meyer Children’s Hospital IRCCS, Florence, 50139 Italy; 2 Department of Life Sciences and Public Health, Pediatric Allergy Unit, University Foundation Policlinico Gemelli IRCCS Catholic University of the Sacred Heart, Rome, Italy; 3 Department of Mother and Child Health, Pediatric Unit, Salesi Children’s Hospital, Ancona, 60123 Italy; 4 Department of Health Sciences, University of Florence, Florence, 50139 Italy; 5 Pediatric Hospital Giovanni XXIII, Pediatric and Emergency Department, AOU Policlinic of Bari, Bari, 70126 Italy; 6 Department of Surgical Sciences, Dentistry, Gynecology and Pediatrics, Pediatric Unit, University of Verona, Verona, 37126 Italy; 7 Pediatric Department, Latisana-Palmanova Hospital, Azienda Sanitaria Universitaria Friuli Centrale, Udine, 33100 Italy; 8 Department of Clinical, Surgical, Diagnostic and Pediatric Sciences, University of Pavia, Pavia, 27100 Italy; 9 Pediatric Clinic, Fondazione IRCCS Policlinico San Matteo, Pavia, 27100 Italy; 10 Translational Research in Pediatric Specialties Area, Division of Allergy, Bambino Gesù Children’s Hospital, IRCCS, Rome, 00165 Italy; 11 Department of Pediatrics, Allergy Unit, AOU Policlinico Gaetano Martino, Messina, 98124 Italy; 12 Department of Woman, Child and General and Specialized Surgery, University of Campania “Luigi Vanvitelli”, Naples, 80138 Italy

**Keywords:** Fatal anaphylaxis, Food allergy, Drug allergy, Prevention, Epinephrine

## Abstract

Anaphylaxis is a life-threatening reaction characterized by the acute onset of symptoms involving different organ systems and requiring immediate medical intervention. The incidence of fatal food anaphylaxis is 0.03 to 0.3 million/people/year. Most fatal food-induced anaphylaxis occurs in the second and third decades of life. The identified risk factors include the delayed use of epinephrine, the presence of asthma, the use of recreational drugs (alcohol, nicotine, cannabis, etc.), and an upright position. In the United Kingdom (UK) and Canada, the reported leading causal foods are peanuts and tree nuts. In Italy, milk seems to be the most common cause of fatal anaphylaxis in children < 18 years. Fatal food anaphylaxis in Italian children and adolescents almost always occurs outside and is characterized by cardiorespiratory arrest; auto-injectable adrenaline intramuscular was available in few cases. Mortality from food anaphylaxis, especially in children, is a very rare event with stable incidence, but its risk deeply impacts the quality of life of patients with food allergy and their families. Prevention of fatal food anaphylaxis must involve patients and their families, as well as the general public, public authorities, and patients’ associations.

## Introduction

Anaphylaxis is a life-threatening reaction characterized by the acute onset of symptoms involving different organ systems and requiring immediate medical intervention [1], which can affect people of any age and can be caused by many types of allergic triggers or, in some cases, can occur without an identifiable cause [1, 2]. The presentation of anaphylaxis is variable, ranging in severity and manifestation, and it usually includes cutaneous, respiratory, gastrointestinal, neurological, and cardiovascular symptoms.

Anaphylaxis-related epidemiological data can differ widely, and variations depend on the definitions used, the study methodology, and the geographical areas in which it occurs. For instance, European data have indicated incidence rates for all-cause anaphylaxis, ranging from 1.5 to 7.9 per 100 000 person/year, with an estimation that 0.3% (95% CI 0.1–0.5) of the population will experience anaphylaxis at some point during their lifetime [3]. It is estimated that 1 in every 3000 inpatients in United States (US) hospitals suffers from an anaphylactic reaction [4].

Fatal anaphylaxis is fortunately quite rare, with a mortality rate of less than 1 death per million inhabitants per year [5–9]. Mortality is estimated at 0.05–0.51 per million people per year for drug-induced anaphylaxis, at 0.03–0.32 for food-induced anaphylaxis, and at 0.09–0.13 for venom-induced anaphylaxis [10–12]. Accurate anaphylaxis mortality data are, however, hampered by the limited recognition of this condition among health professionals, the absence of historical details from eyewitnesses, incomplete death-scene investigations, paucity of specific pathologic findings during the postmortem examination, and the under-notification of anaphylaxis [13–15].

### Causes of fatal anaphylaxis

Drugs are the most common cause of fatal anaphylaxis in many countries [9, 14, 16–20] (Table 1).

**Table 1:** Studies on fatal anaphylaxis

Study	Nationality/Database	Analysis	N° subjects/Mean age	Causes of death (%)	Study period
Liew et al [9] (2009)	Australia/National database	Retrospective case review based on ICD-10 codes on death certificates	112/Not reported	Probable drugs (38), drugs (20), insect (18), food (6), undetermined (13)	8 years (1997–2005)
Tanno et al [14] (2012)	Brazil/Brazilian Mortality Information System	Population-based epidemiologic study using ICD codes on death certificates	498/Not reported	Drugs (42), insect (35), unspecified (21), food (2)	3 years (2008–2010)
Jerschow et al [18] (2014)	USA/United States Vital Statistic Data	Population-based epidemiologic study using ICD CM diagnostic codes on death certificates	2,458 / > 50 years (cause -reported)	Drugs (58.8), unspecified (19.3), venom (15.2), food (6.7)	11 years (1999–2010)
Turner et al [16] (2015)	UK /UK Office on National Statistics database	Hospital admission and fatalities caused by anaphylaxis. Data on anaphylaxis cross-checked against a prospective fatal anaphylaxis registry based on ICD-9 and ICD-10	480/The mean age of fatal food-induced cases ofanaphylaxis was 25 years; The mean age of fatal cases of anaphylaxis due to iatrogenic causes was 58 years; The mean age of fatal cases by sting-induced anaphylaxis was 59 years	Drugs (54.8), food (25.8), insect (19.3)	20 years (1992–2012)
Bilò et al [17] (2020)	Italy/National Register of Causes of Death database, managed by the Italian National Institute of Statistics	Descriptive study An analytical method was developed to identify all the ICD-10 codes related to anaphylaxis deaths, which were divided into two classes: "definite anaphylaxis deaths” and “possible anaphylaxis deaths”	392 definite anaphylaxis deaths and 220 possible anaphylaxis deaths were recorded/ Not reported	Drugs (73.7), unspecified causes (20.7), Hymenoptera stings (5.6)	14 years (2004–2016)
Xu et al [19] (2014)	Canada/Data from the Ontario Coroner’s database	Retrospective case-series analysis of all causes of anaphylaxis-related deaths using data from the Ontario Coroner’s database	92 / Mean age 46.5 years [age range 9 to 86 years]	Food (43), insect (33), drugs (17), unclear (7)	26 years (1986–2011)
Poussels et al [20] (2023)	France/Allergy-Vigilance® Network	Retrospective analysis	25 / Not reported	Food (76), drugs (14), Hymenoptera (12)	19 years (2002–2020)

In a study of anaphylaxis-related mortality coming from an official database of the whole Italian population [17], 392 definite anaphylaxis deaths and 220 possible anaphylaxis deaths (7 of which were children) were recorded in 12 years. The cause of death referred to in this study is the underlying trigger of anaphylaxis and was selected according to the rules and provisions of the tenth revision of the International Classification of Diseases (ICD-10) provided by the World Health Organization (WHO). The average mortality rate for definite anaphylaxis from 2004 to 2016 was 0.51 per million people per year. Definite fatal anaphylaxis was mostly due to the use of drugs (73.7%), followed by unspecified causes (20.7%) and Hymenoptera stings (5.6%). Surprisingly, no cases of fatal anaphylaxis from food were found. This, as reported by the same authors (AA), was probably due to an incorrect coding of food anaphylaxis deaths. On the other hand, some reports in which ICD codes were not used suggested that in some countries, food allergies may be a leading cause of fatal anaphylaxis [21].

In Canada, a retrospective case-series analysis of all causes of anaphylaxis-related deaths using data from the Ontario Coroner’s database between 1986 and 2011 [19] found 92 deaths in the last 26 years. Causes of death, in order of decreasing frequency, included food (40 cases), insect venom (30 cases), iatrogenic (16 cases), and idiopathic (6 cases). No data on pediatric age were reported.

In a recent study from France network vigilance [20], 25 deaths (13 were in children < 18 years) were identified in the 2002–2020 period. Causes of death were food (19 cases), drugs (3 cases), and insects (3 cases). The patients who died were younger (25.6 vs. 40.8 years; *p* = 0.01) than the survivors and mostly presented bronchospasm (56% vs. 29%; *p* = 0.05).

Therefore, the role of foods in fatal anaphylaxis could be reevaluated.

### Incidence of food-induced fatal anaphylaxis

In population-based studies, the rate of fatal anaphylaxis triggered by foods ranges from 0.03 to 0.3 million/people per year. The reported leading causal foods were peanuts and tree nuts [10] (Table 2).

**Table 2:** Studies on the triggers and risk factors of food fatal anaphylaxis

Study	Nationality/Database	Analysis	Time period	Total deaths	Rate of fatal food (per million/year)	Age	Causal foods	Risk factors
Mullins et al [22] (2016)	Australia/Data collected from the Australian Bureau of Statistics (ABS) 1997–2013 and the National Coronial Information System (NCIS) 2000- 2013	Retrospective	1997–2013 2000–2013	324 anaphylaxis fatalities in the period 1997–2013, food (*n* = 23)	1997:0 2014:0.09	Median age 28 (range 4–66)	Seafood 50%, nuts 32%	Known food allergy 91%, asthma 68%, alcohol or recreational drugs 27%, upright posture 68%, delayed use of epinephrine
Xu et al [19] (2014)	Canada/Data from the Ontario Coroner’s database	Retrospective	1986- 2011	92 total, food in 40(43%)	1986:0.32 2011:0.08	Mean age 32 (range 9–78)	Peanuts, tree nuts (55%)	Known food allergy, delayed use of epinephrine
Turner et al [16] (2015)	UK/Data from national databases recording hospital admissions and fatalities caused by anaphylaxis in England and Wales	Retrospective	1992–2012	480 total, food 124(26%)	1992:0–10 2012:0–12	Mean age 25, median age 20. Range (4–85)	Peanuts or tree nuts (73%)	Known food allergy, asthma 78%, change in posture

Recent studies suggest an increase in non-fatal food anaphylaxis [6, 16, 19], but there is no increase in fatalities, except those reported in one Australian study [23].

On the other side, in a UK analysis of national data from 1998–2018, the case fatality rate decreased from 0.7% to 0.19% for confirmed food-induced fatal anaphylaxis [24].

### Risk factors of food-induced fatal anaphylaxis

In infants and young children, food-induced fatal anaphylaxis is very rare, although this age group has the highest rates of anaphylaxis [6, 22].

Most food-induced fatal anaphylaxis occur in the second and third decades of life, without a clear explanation. It may be attributed in part to increased risk-taking behavior and lack of available epinephrine. The identified risk factors include the delayed use of epinephrine [25], the presence of asthma, the use of recreational drugs (alcohol, nicotine, cannabis, etc.), and the upright position. There are many reasons for the delayed use of epinephrine, including lack of knowledge (missed diagnosis, improper technique, etc.), lack of access (epinephrine unavailable, epinephrine never obtained, etc.), or lack of use despite availability. The delayed use of epinephrine is the risk factor most amenable to modification, and increasing efforts are needed to provide epinephrine autoinjectors to all subjects at risk of anaphylaxis. Nevertheless, fatal reactions can occur despite timely administration of adrenaline, accounting for up to one‐third of the cases in the UK Fatal Anaphylaxis registry [26].

About 70–75% of subjects who died from food-related anaphylaxis are asthmatics [16, 22], and most cases of fatal food anaphylaxis are characterized by severe respiratory symptoms. Asthma control should, therefore, be optimized in these subjects, although in some studies there is little evidence for an association with poor asthma control or worsening asthma symptoms leading up to the fatal event [16].

The use of alcohol or other recreational drugs may, through disinhibition, increase the likelihood of accidental allergen exposure, mask the early warning signs of anaphylaxis, or suppress physiological responses to hypotension [10]. Upright posture has also been reported as a risk factor [16, 22], suggesting significant cardiovascular compromise. Fatal food anaphylaxis occurs more often in people with a known food allergy. However, prior reactions are not usually severe, and the severity of prior reactions does not appear to be a risk factor for fatal anaphylaxis [27]. Other risk factors that have been proposed, although without consistent evidence, include race (increased risk in African Americans and UK-resident South Asians), allergy to multiple foods, exercise, and intercurrent illness [10].

### Food fatal anaphylaxis in children

In the last years, few descriptive studies have reported pediatric anaphylaxis fatalities, and most of them are small case series (Table 3).

**Table 3:** Trigger foods in fatal anaphylaxis in children

AA	Source/Period	Total N° of Fatal Food	Causal Food
Sampson [28] (1992) USA	-	6	5 peanuts/tree nuts 1 milk/ egg
MacDougal [29] (2002) UK	1990–2000	8	4 milk 2 peanut 1 egg 1 mixed food
Calvani [30] (2006) Italy	Ministerial data/2000–2002	1	Not specified
Bock [31] (2007) USA	Registry kept by members of the American Academy of Allergy, Asthma & Immunology/2001–2006	17	12 peanuts/tree nuts 4 milk 1 not specified
Levi [32] (2012) Israel	All medical and media data reported 2004–2011	3	3 milk
Poussel [33] (2023) France	France/Allergy-Vigilance® Network	13	Not specified

Sampson [28] reported 6 cases of fatal anaphylaxis in children < 6 years. Peanuts or tree nuts were involved in 5 of the 6 cases. All had a previous allergic reaction.

Another study from the UK and Ireland conducted a retrospective search through death certifications at national statistics offices for fatalities in children 0–15 years from 1990 to February 1998, along with a prospective survey of fatal and severe reactions from March 1998 to February 2000 via the British Paediatric Surveillance Unit. Fatal food-induced anaphylaxis was reported in 8 cases. Milk was the cause of fatal anaphylaxis in 4 children [29].

Calvani et al. [30] found only 2 cases of fatal anaphylaxis in children (1 due to food allergy) in the 2000 – 2002 period using ministerial data.

Bock [31] reported 31 fatalities caused by anaphylactic reactions to food between 2001–2006 in a registry kept by members of the American Academy of Allergy, Asthma & Immunology and The Food Allergy and Anaphylaxis Network. Peanuts/tree nuts were responsible in 12 and milk in 4 of the 17 fatalities in subjects <  = 18 years.

Levi et al. [32] described 4 cases of food allergy-related mortality that were known to medical personnel or were published in the Israeli national communications media in an 8-year period: 3 cases were due to cow's milk (all in children), and 1 adult case was due to hazelnut. All episodes occurred outside (party, restaurant, school, bakery). All 3 children were exposed to a hidden/non-obvious milk allergen (quantities of several mg in 2 cases and 180 mg in 1). All four had a history of asthma but were not on controller medications, and none had experienced previous non-life-threatening accidental reactions. Food anaphylaxis in the UK: analysis of national data, 1998–2018 [24] considered 101 891 people admitted to hospital for anaphylaxis. Food-induced anaphylaxis was identified as the probable cause in 152 deaths. At least 46% of deaths were triggered by peanut or tree nuts (86 of 187, including 35 deaths in 1992–98). Cow’s milk was responsible for 17 of 66 (26%) deaths in school-age children. In school-age children, cow’s milk was considered the most common single cause of fatal anaphylaxis, despite allergy to cow’s milk being uncommon in older children and adults [20].

Poussel [33], in a descriptive national-based study using mortality data routinely reported to the National Mortality Center (CEPIDC) for the years 1979–2014 and extracted on January 31, 2017, identified 25 cases of fatal food anaphylaxis in France; 13 were in children < 18 years, and in one case, the fatal reaction occurred during a hospital oral food challenge. Another case of fatal anaphylaxis was previously reported in a 3-year-old child during a baked milk challenge test [34]. Moreover, 1 fatal reaction in an asthmatic child after eating a small bit of food (baked milk) as part of a desensitization therapy was reported by Canadian media in 2021 (https://www.allergicliving.com/2021/12/20/girl-with-milk-allergy-dies-of-severe-reaction-related-to-desensitization/). Therefore, fatal anaphylaxis occurs after an unnoticed ingestion of allergenic food and exceptionally during diagnostic or therapeutic management of a food allergy. According to these data, the most frequent food allergens involved in fatal anaphylaxis in children are peanuts and tree nuts in the US and milk in the UK and Israel (Table 3).

In the UK, it was found that over the past 25 years, the proportion of fatalities due to peanuts or tree nuts has fallen (attributed to increased awareness of nut allergies by food businesses) and that cow’s milk was the most common cause of fatal anaphylaxis in children [24].

In Italy, a recent study by Bilò et al [17]. found no food fatal anaphylaxis in children or adults. These surprising data were explained by the fact that fatalities due to food do happen to adults; however, they are often not recorded in national registries due to incorrect coding but only reported in national and local newspapers [17]. Cases of food-induced fatal anaphylaxis in children are very rare but occur (Table 3). We collected 9 cases in children < 18 years published by the Italian media between 2010 and 2023 in Italy. 8/9 cases were due to milk allergy and occurred outside the home. Auto-injectable adrenaline intramuscular (AAI) was available only in 3/9 cases.

The mean age was 14 years. Only 1 case occurred in a child < 12 years of age. In many cases, the autopsy confirmed anaphylaxis as the cause of death (Table 4).

**Table 4:** Cases of fatal food anaphylaxis in children published by the Italian media between 2010 and 2023

Name	Age	Year/Location/City	Allergen Certain (C) / Suspected (S)	Suspected food	Injectable Adrenaline available	Autopsy	Other
DP (M)	16 years	2010 / Restaurant / San Giovanni Rotondo (FG) https://corrieredibologna.corriere.it/notizie/cronaca/2010/9-agosto-2010/diciassettenne-vacanza-mangia-gelato-muore-1703545606375.shtml	Wheat allergy (S), other unspecified food allergies, celiac disease	Ice cream with cereals biscuit	Yes Late and improper injection	YES Not available	Restaurant staff informed of allergies
CW (M)	7 years	2015 / Restaurant/ Ravello (Sa) https://www.salernotoday.it/cronaca/scala-bimbo-inglese-morto-allergia-condanna-ristorante-23-marzo-2021.html	Milk (C)	Homemade (with milk) spaghetti with tomato sauce	Yes EpiPen injected by the mother (nurse)	Yes Anaphylactic shock confirmed	Death in Santobono hospital after 3 days
AB (M)	17 years	2015 / Friend’s house / Gaiole in Chianti (Si)/ https://www.lanazione.it/siena/cronaca/shock-anafilattico-allergia-1.1068788	Milk (C)	Pasta with tomato and cheese	No	Yes Anaphylactic shock confirmed	Previous anaphylactic shock at 11 years
BS (F)	16 years	2018 / Home (Cosenza) https://www.quicosenza.it/news/area-urbana/cosenza/262951-studentessa-muore-a-cosenza-per-un-presunto-shock-anafilattico	Milk (C)	Licorice candy	No	No	Dyspnea, hypotension, urticaria
MVS (F)	12 years	2019 / Pizzeria / Villorba (Treviso) https://www.ilmessaggero.it/italia/morta_choc_anafilattico_villorba_pizza_maria_vittoria_salvadori_allergia-4672915.html	Milk (C)	Pizza	No Adrenaline injected by parents	Yes Anaphylactic shock confirmed	Restaurant staff aware of her allergy
FS (F)	16 years	2019 / Bar / Roma https://www.ilmessaggero.it/italia/ragazza_morta_choc_anafilattico_federica_roma-4231128.html	Milk (S) Other unspecified allergies	Cocktail (rum, whisky, fruit cream, coconut milk)	Not reported	Yes Not available	Not available
AA (M)	17 years	2020 / Friend’s home / Taggia (Imperia) https://www.ilsecoloxix.it/imperia/2020/02/27/news/shock-anafilattico-muore-ragazzo-di-17-anni-a-taggia-1.38523834 news/shock-anafilattico-muore-ragazzo-di-17-anni-a-taggia-1.38523834	Milk (C)	Salami sandwich	No	Coroner confirmed cause of death	Similar episode 1 years before
MQ (F)	13 years	2022/Birthday party with friends (Fondi-Latina) https://www.dire.it/16-04-2022/725179-morta-a-23-anni-dopo-un-panino-al-salame-lesperto-gli-allergici-portino-sempre-adrenalina/	Milk (C)	Salami sandwich	Yes Not sure if used right away back at home	Yes (glottis oedema)	Restaurant staff informed of allergy
CP (F)	16 years	2021/School (Enna) https://www.lasicilia.it/cronaca/la-storia-di-carola-allergica-ai-latticini-morta-a-16-anni-dopo-aver-pranzato-a-scuola-cosa-ha-mangiato-1208030/	Milk (C)	Pasta with ragu sauce	Not reported	Yes Not available	First symptoms at school. Died while waiting for the bus

### Milk as a common cause of fatal anaphylaxis in children

Milk allergy is common in childhood and is characterized by a high rate of resolution (50% of the children have tolerance to cow’s milk by 5 years and 75% by the early teenage years) [35]. Persistent milk allergy has been associated with low dose reactions, larger wheal size on Skin Prick Test (SPT) [36], higher serum IgE levels, and a history of anaphylaxis [37]. The persistence of milk allergy has been associated with particular peptide casein sensitization [38]. Cow’s milk has a relatively high protein content, so very low levels of exposure are sufficient to cause reactions. Some described cases of fatal anaphylaxis occurred for hidden minimal quantities (mg) of milk contamination in the ingested food [32].

In the UK and in Italy, milk seems to be the most common cause of fatal anaphylaxis in children < 18. The increasing number of cases of fatal anaphylaxis due to cow's milk in school-age children and young adults and an association between food anaphylaxis and patient's region is also mentioned in the recent European Academy of Allergy and Clinical Immunology (EAACI) guidelines: Anaphylaxis (2021 update) [1].

### Management of children at risk of food fatal anaphylaxis

Prevention of food fatal anaphylaxis involves patients and their families as well as the general public, public authorities, and patients’ associations.

- Measures involving the general public, public authorities, and patients’ associations.
  1. Developing multiple strategies to improve knowledge of life‐threatening anaphylaxis reactions at both national and international public health levels and improve patient access to care and prevention (through prescription of epinephrine autoinjectors and provision of individualized emergency action plans). Anaphylaxis due to food allergy occurs in schools more than in any other community location [39, 40]. Therefore, it may be helpful to target secondary schools and community settings with educational support to help raise general awareness, empower adolescents to confidently self-manage food allergies, and enable schools to develop protocols to minimize any adverse events that may occur.
  2. Optimizing classification and coding for allergic diseases and anaphylaxis. ICD-10 is reported to be an imperfect tool to encode anaphylaxis [17]. The upcoming implementation of the new ICD‐11 classification of allergic and hypersensitivity conditions will be an opportunity to improve anaphylaxis coding to hopefully obtain more accurate data relating to the number of anaphylaxis deaths and their cause.
- Measures involving patients and their families. The reliable identification of patients at increased risk of fatal food anaphylaxis is not currently possible, but some suggestions can be used to help those more likely to have potentially lethal food anaphylaxis:
  1. Information on the role of comorbidities and cofactors. A personal history of asthma is reported in most anaphylaxis fatalities, but no clear relationship has been demonstrated. However, a good control of asthma is mandatory in subjects with food-induced anaphylaxis. The possible role of physical activity and alcohol consumption as cofactors should also be stressed, especially in adolescents and young people who are at greater risk of food-fatal anaphylaxis. The anticipation of higher-risk situations (meals outside the home, school, restaurants, and trips) is also necessary.
  2. Promotion of recognition of allergies and foods at risk. Most fatal food reactions are caused by peanuts, tree nuts, and seafood; in children < 18 years of age, a persistent cow’s milk allergy is also described in a significant proportion of severe and fatal anaphylaxis reactions, in particular in England and Israel. In Italy, no fatal food anaphylaxis was reported in a recent epidemiological study, but this was probably due to an incorrect coding of food-induced anaphylaxis deaths. Moreover, some cases of fatal food anaphylaxis were published by the Italian media in the same period [17]. In our study, milk was the most common cause of fatal anaphylaxis in children < 18 as reported by Italian media. Exclusion of the offending food from the diet is the usual recommendation, along with adequate informative labeling of prepackaged and non‐packaged foods from a list of notifiable ingredients. In this regard, every effort should be made, as fatal anaphylaxis usually happens when the known offending allergen is ingested in hidden/non-obvious form and outside the home. Oral desensitization should be discussed with the patient or parents. Oral immunotherapy (OIT) is the only active therapy currently available that could modify the anaphylactic risk of patients with food allergy [41]. OIT was suggested for treating patients who do not spontaneously acquire tolerance at 4–5 years [42]. OIT is effective in a significant percentage of cases [43], but 20% of children with cow’s milk allergy (CMA) are reported to discontinue treatment due to the significant side effects [44]. In a study, older age was significantly related to a higher risk of OIT failure in children with CMA over time [45]. Therefore, in children with persistent milk allergy and OIT failure, particular psychological and practical support is needed. Many studies reported significant improvement in quality of life (QoL) in children undergoing low-dose peanut OIT [46] or OIT maintenance [47]. OIT seems to have a positive effect on the daily life of patients as well as their parents [48]. Moreover, OIT is considered a driver of decreased severity of allergic reactions [49]. In any case, OIT should be performed very cautiously and by expert medical staff, as 1 fatal reaction during desensitization is reported (https://www.allergicliving.com/2021/12/20/girl-with-milk-allergy-dies-of-severe-reaction-related-to-desensitization/).
  3. Identification of subjects at increased risk of severe reactions is still uncertain. In a recent meta-analysis, IgE sensitization or basophil activation tests were not good predictors [49]. For some foods, molecular allergology may be useful in predicting higher or lower risk of anaphylaxis, particularly when combined with other potential predictors. For tree nuts, IgE against 2S albumins has been reported to be associated with increased rate of any anaphylaxis [49, 50]. A low certain of evidence for an increased risk of severe reactions in food allergy was found for poorly controlled asthma, delayed/inappropriate treatment, use of beta blockers and ACE inhibitors, exercise, and some specific endotypes such as Lipid Transfer Protein (LTP) sensitization and persisting cow milk allergy. Risk of severe outcomes is greatest in adolescence and young adulthood, but the contribution of risk-taking behavior to severe outcomes is unclear. An absence of prior anaphylaxis does not exclude its future risk [51].
  4. Clear and accurate explanation of recognition of symptoms of anaphylaxis and proper treatment. Indication for the prescription of adrenaline according to EAACI Guidelines 2022 [1]. AAI continues to be underused to treat anaphylaxis. In adolescents, repeated education, and training on the appropriate use of AAI is necessary [51]. As food fatal anaphylaxis is due to cardiorespiratory arrest and occurs often after several minutes and often outside the home, with delay in proper treatment, 2 AAI should be absolutely prescribed. Particularly in children with persisting cow milk allergy, if the allergen is ingested, AAI should probably be injected earlier than usually recommended [52]. There is also the need to promote the provision of AAI in ambulances and first aid services [53].

## Conclusions

In conclusion, mortality from food anaphylaxis, especially in children, is a very rare event with stable incidence, but it deeply impacts the quality of life of patients with food allergy and their families. In Italian children < 18 years old, milk seems to be the cause of most cases of food-fatal anaphylaxis. In general, all efforts should be made to reduce the risk of fatal food anaphylaxis through general national and international strategies and optimized preventive and medical care in order to choose the best approach for the single patient.

## Data Availability

Not applicable.

## Abbreviations

UK: United Kingdom
US: United States
ICD-10: International Classification of Diseases
WHO: World Health Organization
AA: Authors
CEPIDC: National Mortality Center
AAI: Auto-injectable Adrenaline Intramuscular
SPT: Skin Prick Test
EAACI: Academy of Allergy and Clinical Immunology
OIT: Oral immunotherapy
CMA: Cow’s milk allergy
QoL: Quality of life
LTP: Lipid Transfer Protein
